# The Hospitalist-Oncologist co-ManagemEnt (HOME) system improves hospitalization outcomes of patients with cancer

**DOI:** 10.1186/s12913-023-10375-0

**Published:** 2023-12-06

**Authors:** Sun-wook Kim, Jung Hun Ohn, Nak-Hyun Kim, Eun Sun Kim, Yejee Lim, Jongchan Lee, Hye Won Kim, Jiwon Ryu, Hee-Sun Park, Koung Jin Suh, Ji-Won Kim, Jin Won Kim, Se Hyun Kim, Yu Jung Kim, Keun-Wook Lee, Jee Hyun Kim, Jong Seok Lee, Hak Chul Jang

**Affiliations:** 1 Department of Internal Medicine, Seoul National University Bundang Hospital, 173-82, Gumi-ro, Bundang-gu, Seongnam, Gyeonggi-do 13620 Republic of Korea; 2 Hospital Medicine Center, Seoul National University Bundang Hospital, Seongnam, Republic of Korea; 3 Department of Internal Medicine, College of Medicine, Seoul National University, Seoul, Republic of Korea; 4 Division of Hematology and Oncology, Department of Internal Medicine, Seoul National University Bundang Hospital, Seongnam, Republic of Korea

**Keywords:** Hospitalist, Hospital medicine, Quality improvement, Delivery of health care

## Abstract

**Background:**

The hospitalist system has been introduced to improve the quality and safety of inpatient care. As its effectiveness has been confirmed in previous studies, the hospitalist system is spreading in various fields. However, few studies have investigated the feasibility and value of hospitalist-led care of patients with cancer in terms of quality and safety measures. This study aimed to evaluate the efficacy of the Hospitalist-Oncologist co-ManagemEnt (HOME) system.

**Methods:**

Between January 1, 2019, and January 31, 2021, we analyzed 591 admissions before and 1068 admissions after the introduction of HOME system on January 1, 2020. We compared the length of stay and the types and frequencies of safety events between the conventional system and the HOME system, retrospectively. We also investigate rapid response system activation, cardiopulmonary resuscitation, unplanned intensive care unit transfer, all-cause in-hospital mortality, and 30-day re-admission or emergency department visits.

**Results:**

The average length of stay (15.9 days vs. 12.9 days, *P* < 0.001), frequency of safety events (5.6% vs. 2.8%, *P* = 0.006), rapid response system activation (7.3% vs. 2.2%, *P* < 0.001) were significantly reduced after the HOME system introduction. However, there was no statistical difference in frequencies of cardiopulomonary resuscitation and intensive care unit transfer, all-cause in-hospital morality, 30-day unplanned re-admission or emergency department visits.

**Conclusions:**

The study suggests that the HOME system provides higher quality of care and safer environment compared to conventional oncologist-led team-based care, and the efficiency of the medical delivery system could be increased by reducing the hospitalization period without increase in 30-day unplanned re-admission.

**Supplementary Information:**

The online version contains supplementary material available at 10.1186/s12913-023-10375-0.

## Introduction

Every year, approximately 2.8 million adult hospitalizations in the United States are related to cancer and there are 1.0 million hospital admissions with cancer as the principal diagnosis [1]. Hospital admission principally for cancer cost $23.0 billion, accounting for 6.2% of the $372.6 billion aggregate adult hospital costs in 2017. The situation is similar in Korea; approximately 408 thousand cancer-related admissions occur every year, and the associated cost is approximately $5.0 billion [2].

The primary objective of admissions associated with cancer predominantly revolves around providing supportive care. The intricate nature of supportive care for cancer patients necessitates a comprehensive understanding not only of specialized oncology but also of general medicine and human pathophysiology [3]. Especially, the complexity of care runs into an extreme in inpatients’ supportive care. Inpatient care delivery teams operate at the intersection of basic biology, clinical medicine overlaid several departments, patients’ or family members’ concerns, and their emotional needs; [4] the teams must review the diagnosis process, organize the history of cancer treatment and response, assess current problems, and make a diagnostic or treatment plan considering patients’ comorbidities, preferences, familial support, and financial status simultaneously.

The reorganization of inpatient services has led to the emergence of the hospitalist model, which involves specialized general physicians who focus on providing high-quality, safe, and cost-efficient care for patients during their hospital stay [5]. Hospitalists possess expertise in managing complex medical conditions and are particularly adept at caring for patients with multiple morbidities. While the history of medicine has witnessed the creation of specialized and subspecialized fields based on anatomical organs or physiological systems, the involvement of numerous specialized physicians in a patient’s care can inadvertently lead to communication gaps and coordination challenges, particularly in inpatient supportive care [6]. Furthermore, as older patients often have frailty and increasingly complex multimorbidity, the demand for hospitalists who can understand and manage these patients is also growing.

Hospitalists play a crucial role in improving hospital system performance by delivering comprehensive care to complex patients with diverse medical needs [7]. They also support initiatives to reform the healthcare delivery system, promote patient safety, reduce hospital stay length, and decrease re-admissions. Previous studies have shown that hospitalist systems provide safe and cost-effective care, leading to better outcomes and patient satisfaction, especially for complicated patients [8–10].

Hospitalists working in an oncology ward require specialized skills and knowledge to provide care for complex cancer patients, as well as close communication with various healthcare professionals such as oncologists, radiologists, pharmacists, nutritionists, and social workers. This specialized approach allows for effective acute hospital care that takes into account the patient’s individual cancer treatment plan [11]. Nevertheless, the implementation of the hospitalist system in oncology wards remains uncommon [12]. Therefore, we conducted a pre-post study to evaluate the impact of adopting the hospitalist system in a supportive oncology ward.

## Methods

### Study design

We designed a retrospective pre- and post-study to compare the management of cancer patients who require supportive care before and after the involvement of hospitalists.

### Hospitalist-Oncologist co-ManagemEnt (HOME) system

On January 1, 2020, a hospitalist service model in the 32-bed supportive cancer care ward was established at the Hospital Medicine Center at the Seoul National University Bundang Hospital (SNUBH), a 1300-bed teaching hospital in Korea. The goals of the system were to provide comprehensive care for hospitalized patients with cancer while implementing quality-based practice improvement. Cancer patients are admitted to the oncology ward, which operated both before the conventional system and after the HOME system implementation, either through outpatient oncology clinics or Emergency Rooms (ERs), irrespective of the primary cancer site. We excluded the following patients with any contraindications: those with hemodynamic instability requiring critical care facilities (such as Ventilator Care, ExtraCorporeal Membrane Oxygenation, and Continuous Renal Replacement Therapy), those primarily requiring end-of-life care, and those with hematologic malignancy [13]. We excluded hematologic malignancy patients because inpatient care of hematologic malignancy required more hematology-specific care than generalist care that hospitalists provide.

Before operating the HOME system, two internal medicine residents who were directed by patients’ attending oncologists cared primarily for the daytime and night shift. The attending oncologists made ward rounds daily with the trainees on work time to take information about present illness, examine the patients, and make a diagnosis or treatment plan.

However, under the HOME system, hospitalists are in charge of the main care for patients’ various problems and needs, including the chief complaint of hospital admission. When a patient is admitted, the hospitalist reviews the patient’s reason for admission and medical history/treatment records, performs an examination, and establishes a diagnosis and treatment plan for active problems. They have autonomy to assess symptoms and signs, evaluate test results and treatment effectiveness, and establish discharge plans. They can make important clinical decisions about general medical care on their won and can focus on the treatment of hospitalized patients more than oncology staffs who need to balance outpatient care. Additionally, they discuss with the attending oncologists regularly about detailed past history not recorded in medical records, remaining problems, and further anticancer treatment options available in the current situation. Two hospitalists manage the inpatients in the daytime from 7:30 am to 6:00 pm on weekdays and 7:30 am to 12:30 pm on weekends, and the night shift is assigned to internal medicine residents.

### Patient selection and data collection

All patients who were admitted to the oncology supportive care ward from January 1, 2019, to January 31, 2021, were included in the study. As the HOME system introduced on January 1, 2020, we assumed January 2020 for period of implementation. Baseline patient characteristics, including age, sex, height, weight, type of cancer, and reason for hospitalization, were collected from electronic medical records (EMR). The primary reason for hospitalization was classified by cancer progression-related complications (e.g., metastasis, gastrointestinal tract obstruction, respiratory tract obstruction, bleeding, pleural or pericardial effusion, ascites, or hypercalcemia), diagnosis or re-evaluation (e.g. staging work up, assessment of treatment response), infection (e.g., neutropenic fever, pneumonia, urinary tract infection, cholangitis, and intra-abdominal abscess), cancer treatment (e.g. intravenous or intrathecal chemotherapy, gamma knife surgery), supportive care for pain control or nutritional support, and complications of anticancer treatment (e.g., drug-induced pneumonitis/hepatitis/colitis, nausea, vomiting, and diarrhea) (Supplementary Table 1). For patients with two or more reasons for hospitalization, the round-table discussion was held with all authors who were board-certified internists and working in the hospital medicine center to determine the main reason of each case in depth. In the SNUBH, the registered nurse evaluates patients’ risk of falls, bedsores, and delirium regularly from admission as usual care. In this study, the scores of the Henrich II Fall Risk Model, Braden Scale, and Nu-DESc acquired on the first day of admission were collected [14].

### Rapid response system (RRS) and SNUBHian alert-system for errors (SAFE) report

Approximately 60–84% of in-hospital Cardio-Pulmonary Arrests (CPAs) present with abnormal clinical signs before the sudden events [15]. To detect early and correct preventable causes of CPA, the RRS, which is separate from the traditional CPA team, has been running since October 2012 in SNUBH. RRS primarily relies on the EMR screening system and encompasses 10 triggering variables, including vital signs obtained by nurses or automated monitors, as well as laboratory results. Each variable is assigned a predetermined threshold for activation (Supplementary Table 2) [16]. The RRS team is composed of critical care specialists with experience in respiratory medicine, thoracic surgery, emergency medicine, and specialized nurses. After activation of RRS, RRS-charging nurses and physicians intervene for alert-listed patients. They review the patients’ medical records, examine the patients, discuss the cause of current problems, and make a further investigation and treatment plan, including transfer to an intensive care unit (ICU) [17].

SNUBH also collect safe report called SAFE to reduce the same errors in the future, to reform the process that has a potential risk, and to improve the quality of hospital services. Medical staff must report medication errors, blood transfusion errors, fall down, errors in medical or surgical procedures, self-harm or suicide, medical device malfunction, and environmental issues after the accidents [18]. The authors reviewed all RRS records and SAFE reports of the study participants, which were documented during the index hospitalization.

### Study outcomes

The primary outcome was the total length of stay. The secondary outcomes were the occurrence and type of SAFE events, RRS activation, CPR, unplanned ICU transfer, all-cause in-hospital mortality, and 30-day unplanned re-admission or ER visits.

### Statistical analysis

Continuous variables were reported as means (SDs) and analyzed using an unpaired *t*-test for normally distributed data and the Mann-Whitney U test for non-normally distributed data. Categorical variables were expressed as counts and percentages, and their proportions were compared using the chi-square test or Fisher’s exact test. Due to the potential heterogeneity between the two groups, which could serve as confounding variables, we employed multivariable linear or logistic regression analysis to adjust. Differences were considered statistically significant at *P* < 0.05, and all analyses were 2-tailed. It was determined that 393 participants would need to be included in each group for 80% power to detect a significant difference in the primary outcome, LOS, by 2 days with 2-sided α of 0.05. Sample size was calculated at https://www.sample-size.net/ [Accessed 19 November 2023]. Variables not following normal distributions were log transformed. We analyzed data using SPSS 26.0 (IBM SPSS Statistics, Armonk, United States of America) software and R v4.0.2 (https://www.r-project.org/).

## Results

**Fig. 1:**
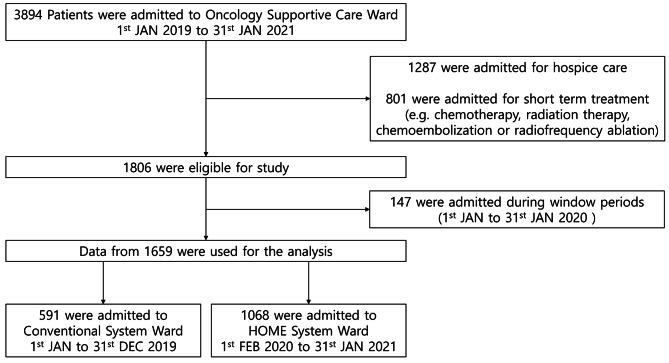
Flow of patients through study

Between January 1, 2019, and January 31, 2021, a total of 3,894 patients were admitted to the single supportive care oncology ward at SNUBH, which was transformed into the HOME system on January 1, 2020. Among these patients, 1,287 patients admitted for hospice care or hospice transitioning, and 801 patients hospitalized for short-term (≤ 3 days) cancer evaluation or treatment (including percutaneous biopsy, chemotherapy, radiation therapy, chemoembolization, or radiofrequency ablation) were excluded. Additionally, we excluded 147 patients admitted from January 1, 2020, to January 31, 2020, which was the window period for setting up the HOME system. Finally, data from 1659 patients were used for the analysis (Fig. 1).

The baseline demographic, laboratory, and oncologic characteristics of all participants are presented in Table 1. There were more men in the HOME system group, and they were taller and had higher BUN and creatinine concentrations. The proportion of lung and urinary tract cancer patients was higher in the HOME system than in the conventional system, and more patients were hospitalized for complications due to cancer progression rather than cancer treatment, in line with the purpose of establishing the HOME system that focused on supportive care in cancer. There was no significant difference in patients’ risks of bedsores and delirium between both groups. However, the risk of falls was higher in the HOME system group.

**Table 1:** Demographic, Laboratory, and Oncologic Characteristics of Study Population

	Conventional System Group (n = 591)	HOME System Group (n = 1,068)	*P* values
**Demographic**			
Age, y	62.7 (12.94)	63.8 (12.19)	0.102
Sex, male/female	221/370	595/473	***< 0.001***
Height (cm)	160.0 (7.71)	161.3 (8.78)	***0.003***
Weight (kg)	57.2 (11.66)	57.7 (11.16)	0.374
Body mass index (kg/m^2^)	22.3 (4.10)	22.1 (3.61)	0.382
**Laboratory**			
WBC (x 10^3^/µL)	9.60 (13.30)	8.67 (6.32)	0.433
Hemoglobin (g/dL)	10.0 (1.63)	10.3 (1.95)	0.085
Platelet (x 10^3^/µL)	209.3 (136.43)	232.7 (127.95)	0.071
BUN (mg/dL)	18.1 (12.9)	19.7 (12.9)	***0.016***
Creatinine (mg/dL)	0.78 (0.77)	0.88 (0.88)	***0.039***
Cholesterol (mg/dL)	142.7 (44.9)	143.0 (46.8)	0.924
Protein (g/dL)	6.0 (0.85)	5.9 (0.87)	0.531
Albumin (g/dL)	3.0 (0.57)	3.1 (0.81)	0.072
AST (IU/L)	51.0 (83.1)	56.3 (80.6)	0.228
ALT (IU/L)	32.5 (55.8)	33.8 (65.5)	0.692
**Primary Cancer**			
Lung Cancer	168 (28.4%)	374 (35.0%)	***0.006***
Gastric Cancer	79 (13.4%)	120 (11.2%)	0.207
Breast Cancer	74 (12.5%)	105 (9.8%)	0.099
Colon and Anorectal Cancer	63 (10.7%)	105 (9.8%)	0.611
Hepatobiliary Cancer	42 (7.1%)	69 (6.5%)	0.610
Kidney and Bladder Cancer	22 (3.7%)	76 (7.1%)	***0.005***
Soft Tissue Sarcoma	33 (5.6%)	36 (3.4%)	***0.039***
Ect.	110 (18.6%)	183 (17.2%)	0.460
**Reason for Hospitalization**			
Complication Related to Cancer Progression	166 (28.1%)	360 (33.7%)	***< 0.001***
Diagnosis or Re-evaluation	90 (15.2%)	246 (23.0%)	
Infection	115 (19.5%)	203 (19.0%)	
Cancer Treatment	142 (24.0%)	81 (7.6%)	
Pain Control or Nutritional support	36 (6.1%)	93 (8.7%)	
Complication of Anticancer Treatment	30 (5.1%)	54 (5.1%)	
Ect.	12 (2.0%)	31 (2.9%)	
**Patient Assessment**			
Hendrich II Fall Risk Model	3.8 (2.76)	4.2 (2.77)	***0.001***
Braden Scale	19.6 (2.98)	19.8 (2.94)	0.125
Risk of Delirium (Nu-DESc)	0.12 (0.57)	0.1 (0.53)	0.291

Data are presented as mean (SD) or number (%)

Abbreviations: WBC; white blood cell, BUN; blood urea nitrogen, AST; aspartate aminotransferase, ALT; alanine aminotransferase

The total length of hospital stay (the primary outcome) in the HOME group was about 3 days (18.9% reduction) shorter than that in the conventional group (12.9 vs. 15.9 days, *P* < 0.001) (Table 2). Moreover, even after adjusting for cancer types and reasons for admission, this result still exhibited statistical significance. The incidence of safety events was significantly lower in the HOME system group compared to the conventional group (33/591 vs. 30/1068 events/person, *P* = 0.006) and RRS activation (43/591 vs. 24/1068 events/person, *P* < 0.001) were also significantly reduced in the HOME system group. Considering the length of hospital stay, the HOME system group exhibited a lower incidence of safety events (22.4 vs. 39.4 events/10,000 person*day, *P* = 0.025) and RRS activations (24.6 vs. 70.3 events/10,000 personday, *P* < 0.001) compared to the conventional group. Detailed information is provided in Table 3.

**Table 2:** Comparison of In-Hospital and After Discharge Outcomes by Groups

	Conventional System Group (n = 591)	HOME System Group (n = 1,068)	β or OR (95% CI)	*P* value
**In-hospital**				
Length of Stay (Days)	15.9 (12.94)	12.9 (10.05)	-3.54* (-2.41, -4.67)	< 0.001**
Occurring Safety Event (No of Patients)	33 (5.6%)	30 (2.8%)	**0.48 (0.28, 0.81)**	***0.006***
RRS Activation (No of Patients)	43 (7.3%)	24 (2.2%)	**0.25 (0.15, 0.42)**	***< 0.001***
CPR	5 (0.8%)	5 (0.5%)	0.57 (0.15, 2.12)	0.386
Unplanned ICU Transfer	4 (0.7%)	7 (0.7%)	1.03 (0.30, 4.08)	0.962
All-cause In-hospital Mortality	56 (9.5%)	80 (7.5%)	0.73 (0.51, 1.06)	0.099
**After discharge**				
In 30-day Unplanned Re-admission	99 (16.8%)	137 (12.8%)	0.77 (0.57, 1.03)	0.076
In 30-day ER Visit	106 (17.9%)	181 (16.9%)	0.92 (0.70, 1.21)	0.539

Abbreviation: RRS; rapid response system, CPR; cardiopulmonary resuscitation, ICU; intensive care unit, ER; emergency room

All *P* values are adjusted for cancer type and reasons for admission

*β for untransformed LOS

**P value for log-transformed LOS

**Table 3:** Safety Event and Rapid Response System (RRS) Activation by Groups

	Conventional System Group (person*day = 9,389)	HOME System Group (person*day = 13,818)	*P* value
**Safety Event**	**37 (39.4)**	**31 (22.4)**	***0.025***
Fall Down	17 (18.1)	9 (6.5)	***0.015***
Medication Error	14 (14.9)	10 (7.2)	0.095
Medical or Surgical Procedure Error	1 (1.1)	2 (1.4)	1.00
Transfusion Error	0 (0.0)	1 (0.7)	1.00
Ect.	5 (5.3)	9 (6.5)	0.79
**RRS Activation**	**66 (70.3)**	**34 (24.6)**	***< 0.001***
Respiratory Distress	57 (60.7)	29 (21.0)	***< 0.001***
Shock	3 (3.2)	2 (1.4)	0.40
Arrhythmia	2 (2.1)	2 (1.4)	1.00
Altered Mental Status	3 (3.2)	0 (0.0)	0.07
Metabolic Acidosis	1 (1.1)	1 (0.7)	1.00
Others	2 (2.1)	3 (2.2)	1.00

* Data shown by number of events and number of events divided by 10,000 person*day

The HOME system and conventional group showed no statistically significant differences in CPR (0.5% vs. 0.8%; *P* = 0.290), unplanned ICU transfers (0.7% vs. 0.7%; *P* = 0.866), or all-cause in-hospital mortality rates (7.5% vs. 9.5%; *P* = 0.099), respectively. Additionally, there were no statistically significant differences in rates of unplanned re-admission (12.8% vs. 16.8%; *P* = 0.076) and ER visits within 30 days following the index discharge between the HOME system and conventional groups (16.9% vs. 17.9%; *P* = 0.539).

## Discussion

The main findings of this study are as follows: The length of hospital stay was shortened by 3 days (18.9% reduction compared with the conventional system), and the frequency of RRS activation and safety event, including fall down were decreased after the introduction of the HOME system. These results suggest that hospitalists working in the supportive care oncology ward could evaluate the patients more rapidly and accurately, provide well-designed diagnostic processes and treatment plans, and share the plans with patients, their families, and other house staff. Moreover, they also promoted a safer hospital environment for patients. The reduction in hospitalization duration without a concomitant rise in readmission rates signifies the potential for accommodating a greater number of inpatient care-seeking patients efficiently, thereby decreasing their waiting times, without necessitating the expansion of additional medical resources [19].

As advances in diagnosis and treatment technology improve cancer patient survival, cancer-related hospitalizations are steadily increasing, and complex cases in which several chronic diseases, in addition to cancer, are combined are also rapidly increasing due to the aging of the population [20]. To care for complicated inpatients, rich clinical experience, excellent skill, comprehensive perspective, and intuition are required, and a hospitalist is a good fit for those roles. From the viewpoint of the entire health care system, if those complex cases are not properly managed, they will inevitably have a knock-on effect on other services (e.g., unscheduled hospital visits), even it can cause the system failing, as seen from the recent NHS confrontation [21, 22]. The most significant strength of this study is that with the HOME system, hospitalists could provide effective and safe care for supportive cancer patients with multimorbidity, and they can contribute to the efficient distribution of medical resources [23].

Hospital medicine started in the late 1990s to provide safer and more efficient medical services to hospitalized patients. In Korea, the hospitalist system started as a pilot project in 2015, and as of March 2022, the number of hospitalists had increased to 303. The United States, which started the system much earlier than Korea, had only 10,000 hospitalists in 2003, and the number was rapidly increasing to over 50,000 in 2016. The hospitalists are actively engaged in various fields, such as acute medical units, short-term examination and treatment wards, and general surgical units [24]. Despite the increasing adoption of the hospitalist system in various specialties, there are few studies on the efficacy of hospitalists in oncology wards where continuity of care and the patient-physician relationship are considered critical. Two previous studies about the efficacy of hospitalists managing patients with cancer demonstrated comparable outcomes with respect to quality measures like length of hospital stay and re-admission to those cared for by oncologist-led inpatient services [22, 25]. Notably, our study suggests that inpatient care by hospitalists co-operating with oncologists could be superior in quality and safety measures to that by the conventional system.

Several factors may have contributed to the superior efficacy and safety of the HOME system in our study, such as the precise medical performance, immediate response to sign or test results, effective communication, and interpersonal skills of hospitalists co-operating with other specialists including oncologists, compared to the conventional system. In contrast, primary oncologists, despite building strong patient rapport, encounter constraints due to dividing their focus between outpatient clinics and inpatient responsibilities. However, within the HOME system, hospitalists can allocate more time to provide patients with comprehensive explanations regarding their condition, prognosis, and available treatment alternatives. Moreover, they can offer multi-dimensional care considering chronic diseases, in addition to cancer, and reflecting the preference or socio-economic situation of patients or their families after in-depth interviews. Indeed, some studies indicated that patient satisfaction was higher in inpatient care provided by hospitalists than by primary oncologists [26]. A recent survey also found that oncologists have a favorable perception of hospitalist-led inpatient care for cancer patients, and their acceptance of the hospitalist model is generally high [27].

Our study has several limitations. First, it was a pre-and-post analysis; patients’ primary cancers and reasons for hospitalization were different between the two groups. Despite employing regression analysis to adjust potential confounders that might impact the outcomes, achieving perfect adjustment for all influential factors remained unattainable. The opening of the HOME ward led to the consolidation of cancer patients in need of supportive care who were previously distributed across various wards, resulting in potentially different cancer types and reasons for hospitalization before and after the implementation of the system. Specifically, the care of lung cancer patients with unstable vital signs due to frequent respiratory complications may have been impacted by the HOME system. Nevertheless, there were no significant differences in patients’ frailty, which can be referred to as fall, sore, and delirium risks, between the two groups(Table 1). Throughout the entire (pre and post) study period, there were no systemic changes that could have influenced outcomes, such as changes in nursing staff, apart from variations in the mix of physicians, and no modifications in quality improvement activities were made. In the conventional system group, a higher frequency of patient admissions pertained to cancer treatment, contrasting with the HOME system group, where a predominant reason for admissions revolved around complications related to cancer progression. This echoes the underlying desire of instituting the HOME system, intending Hospitalists to attend to complex patients with multimorbidity. Given that patients requiring supportive care often necessitate longer hospital stays compared to those admitted solely for cancer treatment, it is plausible that the disparity in analyzed lengths of stay may underestimate the true difference. The study’s analysis period coincided with the global COVID-19 pandemic that began in early 2020. Although it is challenging to ascertain whether the pandemic directly contributed to the shorter length of hospital stay and reduced incidence of safety events during hospitalization, there was no in-hospital outbreaks within the HOME ward during the study period. Second, since the study was performed at a single tertiary care institution, the findings might not be generalized to other hospitals with different conditions. However, based on the findings in previous studies on the efficacy of hospitalist systems in other settings, the hospitalization period and cost can be reduced when the hospitalist is in charge of the inpatients in the HOME system compared to the conventional attending physician-resident system [28, 29]. Third, despite the reduction in hospital stays, no discernible change was noted in the 30-day unplanned readmission rate or emergency room visits subsequent to discharge. A study conducted on the re-admission rate in the general medicine hospitalist service at a comprehensive cancer center in the United States also reported a re-admission rate of 22.6% [30]. Notably, unplanned re-admissions were more frequent in patients with metastatic cancer or those afflicted by more than three comorbidities. As the patients managed by the HOME system were mostly admitted for complications from cancer progression, we speculate that the characteristics of the patient group were responsible for the high rate of re-admissions. Moreover, the study found that there was no reduction in emergency department (ED) revisits within 30 days of discharge, warranting further research to identify strategies to decrease these rates and reduce high readmission rates among cancer patients.

Meanwhile, in most cases, the primary care of inpatients was assigned to the residents. In Korea, all citizens have medical insurance operated by the government, and the system adopts fee-for-service payments. Every image workup, blood test, operation, invasive procedure, and medication has a price. Furthermore, newly developed drugs, including immune checkpoint inhibitors, signal or surface-targeted monoclonal antibodies, and signal modulators, are priced very high. However, examining patients’ symptoms and signs, doing physical examinations, checking lab and image test results, reasoning the data logically, counseling the patients, and ordering proper examination or treatment every day in the inpatient ward have been undervalued. All the above works’ price is included in the inpatient accommodation charge, and the cost is fixed, regardless of whether the medical team provides high-quality medical service. Therefore, instead of improving the quality of inpatient care, hospital executives classify hospitalization care as low value-added activity and have the incentive to dedicate most of the hospitalization care, including night duty, to the residents in the name of education. However, as demonstrated by this study, efforts to provide high-quality inpatient care can lead to a reduction in safety incidents, shorter hospital stays, and prevent unnecessary readmissions, thereby contributing to efficient distribution of medical resources at the national level. In terms of hospital management, it should not be overlooked as it can increased the hospital utilization rate by using same resources.

### Electronic supplementary material

Below is the link to the electronic supplementary material.

Supplementary Material 1

## Data Availability

The datasets generated and analyzed during the current study are not publicly available due to the Institutional Review Board of the Seoul National University Bundang Hospital requirement of not publishing the raw data to the public. However, the de-identified raw data are available from the corresponding author (Jung Hun Ohn) on reasonable request.
