# Supplementary material for: The Hospitalist-Oncologist co-ManagemEnt (HOME) system improves hospitalization outcomes of patients with cancer
Source: BMC Health Serv Res. 2023 Dec 6;23:1367. doi: 10.1186/s12913-023-10375-0 (PMC10702015; doi:10.1186/s12913-023-10375-0)
Supplement: Supplementary file 1 — Supplementary Material 1 [file 12913_2023_10375_MOESM1_ESM.docx]

**Supplementary Table 1. Classification of Reason for Hospitalization**

| - Infection   Neutropenic fever  Pneumonia  UTI  Cholangitis  Colitis  Intra-abdominal/perianal abscess  Complications of anticancer treatment  Drug-induced pneumonitis/hepatitis/colitis  Nausea, Vomiting, Diarrhea  Diagnosis or Re-evaluation | Complication related to cancer progression  Metastases  Obstruction  Gastrointestinal tract  SVC syndrome  Respiratory tract  Bleeding  Pleural/Pericardial effusion/Ascites  Hypercalcemia  Pain control, Nutritional support  Cancer treatment |
| --- | --- |

Abbreviation: UTI; urinary tract infection, SVC; superior vena cava

**Supplementary Table 2. Rapid Response System (RRS) Activation Criteria**

| Criteria 1 SBP < 90 mmHg |
| --- |
| Criteria 2 HR <50, >140 /min |
| Criteria 3 RR <10, >30 /min |
| Criteria 4 BT >39, <36 ℃ |
| Criteria 5 SpO_2_ <90% |
| Criteria 6 pH < 7.25 |
| Criteria 7 PaCO_2_ >50 mmHg |
| Criteria 8 PaO_2_ <55 mmHg |
| Criteria 9 Lactic acid > 4 mmol/L |
| Criteria 10 Total CO_2_ <15 mmol/L |

Abbreviation: SBP; systolic blood pressure, HR; heart rate, RR; respiration rate, BT; body temperature
